# A novel implantable device for sensory and affective assessment of orofacial pain in rats

**DOI:** 10.3389/fvets.2022.1028147

**Published:** 2022-10-28

**Authors:** Xiaoling Huang, Zhenxing Li, Jiahui Ma, Dong Huang, Xuebin Yan, Haocheng Zhou

**Affiliations:** ^1^Department of Pain, The Third Xiangya Hospital and Institute of Pain Medicine, Central South University, Changsha, China; ^2^Department of Anesthesiology, Hunan Provincial People's Hospital, The First Affiliated Hospital of Hunan Normal University, Clinical Research Center for Anesthesiology of ERAS in Hunan Province, Changsha, China; ^3^Hunan Key Laboratory of Brain Homeostasis, Central South University, Changsha, China

**Keywords:** orofacial pain, chronic pain, electrical, noxious, stimulation, sensory, affective, IoN-CCI

## Abstract

**Background and objective:**

Orofacial pain, in particular, chronic orofacial pain remains a great challenge in clinical practice. To better understand the underlying mechanism of disease, it is essential to apply a feasible and stable preclinical measurement of facial pain. Here, we introduced a novel electrical noxious stimulator in freely behavioral rodents and examined its validation in both naïve and chronic orofacial pain animals.

**Methods:**

One subcutaneous device of electrical stimulator was implanted in the facial region for delivery of the nociceptive input. The sensory component of orofacial pain was assessed by response scoring tool, and conditioned place aversion (CPA) paradigm for pain affect respectively. To confirm its usage in chronic pain state, the chronic constriction injury of the infraorbital nerve (ION-CCI) model was then applied.

**Results:**

We found that responsive scores increased with stimulation intensity, and acted in a dosage-dependent manner, which can be attenuated by the administration of morphine intraperitoneally. Naïve rats displayed significant aversive reaction to the noxious electrical stimulation (25V) in the CPA testing. In addition, an obvious sensory hypersensitivity to electrical stimulation was confirmed by the increased response scores in ION-CCI rats. Furthermore, ION-CCI animal showed significant avoidance to electrical stimulation at relatively low intensity (10V), which was innoxious to naïve rats.

**Conclusion:**

Our findings may provide an alternative pre-clinical measurement of orofacial pain, to quantitively assess both sensory and affective component of orofacial pain.

## Introduction

Results from the international survey indicated that orofacial pain occurs about one in ten adults, and is more commonly affected in the female gender (1). More recently, it has been estimated that the prevalence of orofacial pain is 1.9% in the humans aged between 40 and 69 in the UK, about half (48%) of whom reported chronic pain state (2, 3). Compared with body pain, painful experience originates from facial region is generally ranked more severe and emotionally suffering, potentially augmented by specific supraspinal neural circuits (4). One common and characteristic phenotype of chronic orofacial pain is trigeminal neuralgia, which has been described as one of the most painful diseases in human (5). In addition to pain severity, co-morbidities such as depression, anxiety, and sleep disorder may also contribute to a significant reduction of quality of life (6, 7). However, current management of chronic orofacial pain remains a great challenge to pain physician, due to limited knowledge of its mechanism.

To better understand the underlying mechanism, one suitable and relevant animal model is essential. Currently, chronic constriction injury of infraorbital nerve (ION-CCI) is the most widely used animal model in this field (6). To capture the clinical feature, an asymmetric face grooming will assess the non-evoked or spontaneous pain-like behavior. Besides, the characteristic patterns of spontaneous pain-like grooming can also be distinguished in rats with inflammatory facial pain (8). In addition to spontaneous episodes, another featured hallmark of chronic orofacial pain is its abnormal reaction to noxious and non-noxious stimuli.

To induce the evoked-behavior, mechanical stimuli can be conducted to the trigeminal territory with Von Frey filaments (6). Following facial stimulation, distinct elements are systematically identified and recorded for the calculation of the response score (6, 9–11). In trigeminal neuropathic pain model, both hypo- and hyper responsiveness can be observed in the ION-CCI region (6). However, the mechanical stimulation must be delivered manually to the orofacial region in the operant behavioral assay, which remains a great challenge to approach the testing probes to the target of the face. Similarly, thermal assessment is a much more complicated measurement of orofacial pain, especially for the mice species (12). One potential solution is to apply one air-puff device with fixed distance and angle to the stimulation site (13, 14). Current methods of stimulus-evoked behavior is generally trigged by the external stimuli, yet internal nociceptive mechanism may also reflect the physiological processing of orofacial pain.

The implantable device may provide an alternative option for evoked-painful stimulation. It has been demonstrated that the implantation of electrical stimulation in the dura mater can induce the isolated grooming and head-flick activity in a frequency-dependent manner (15). Compared with thermal and mechanical stimuli, one advantage of electrical nociception is its adjustable and stable output (16). Additionally, the design of implantable device makes it feasible to conduct the operant behavior paradigms, which can be applied to evaluate the affective component of orofacial pain (12). However, few study focused on the validation of implantable electrical device for orofacial evoked-pain. In this study, we introduce one novel implantable electrical stimulation device, and we aim to investigate its usage for orofacial pain delivery in both naïve and trigeminal neuropathic pain rats.

## Methods and materials

### Animals

**Figure 1:**
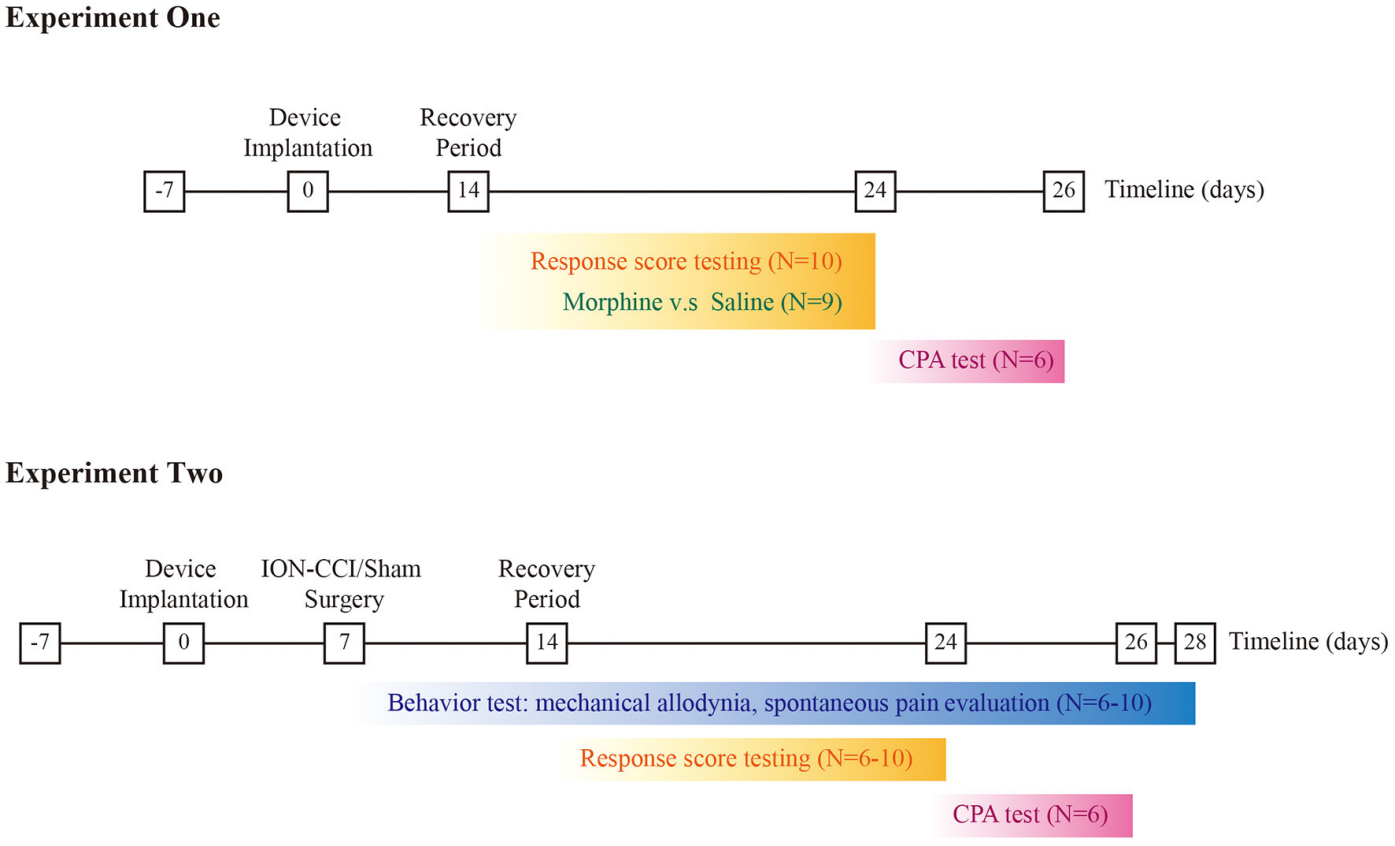
Schematic of experimental design. Top panel refers to the experiment one, and the bottom panel for the experiment two respectively.

The protocol of animal experiment was approved by the Institutional Animal Care and Use Committee (NO. 81901146), Central South University and were consistent with Regulations for the Administration of Affairs Concerning Experimental Animals (Science and Technology Commission of China) to guarantee the welfare of experimental animals. Male Sprague-Dawley rats were obtained from Hunan SJA Laboratory Animal CO., Ltd, Changsha, Hunan Province, China. The animals were kept at YOUCHENG Bio-Services Facility, with controlled humidity (55–60%), room temperature (22–26°C). Food (crude protein percentage >18%) and water were supplied in home cage, with one light-dark cycle set between 8:00 AM and 8:00 PM. The experimental subjects were weighted 250–280 g when arrived and habituated one week before the onset of experiment. The weight of experimental subjects ranged between 320 and 350 prior to procedure. The schematic of experimental design is given in the Figure 1.

### Drugs

To evaluate the effect of analgesic agents on response scoring testing (Figure 1), we applied 10 mg kg^−1^ morphine hydrochloride (First Shenyang Pharmaceutical Co. Ltd.) intraperitoneally in the morphine group (0.5 ml) prior to the electrical stimulation, and 0.5 ml saline was injected intraperitoneally in the control group retrospectively.

### ION-CCI model

The procedure of ION-CCI model was conducted as described previously (17). Specifically, rats were pre-anesthetized by inhalation in a sevoflurane (6–8%) anesthesia induction chamber for 1 minute, followed by maintenance of anesthesia with a sevoflurane (1.5–2.0%) mask. The skin between the eye and whisker pad was incised. One 0.5 cm incision was then made for exposure of the distal segment of the ION. Following the exposure of ION, the distal branch of ION was properly and loosely tied with two chromic catgut knots, at a distance of 2 mm (17, 18). The skin was then closed with a polyester suture (4–0).

### Stimulation electrode construction

**Figure 2:**
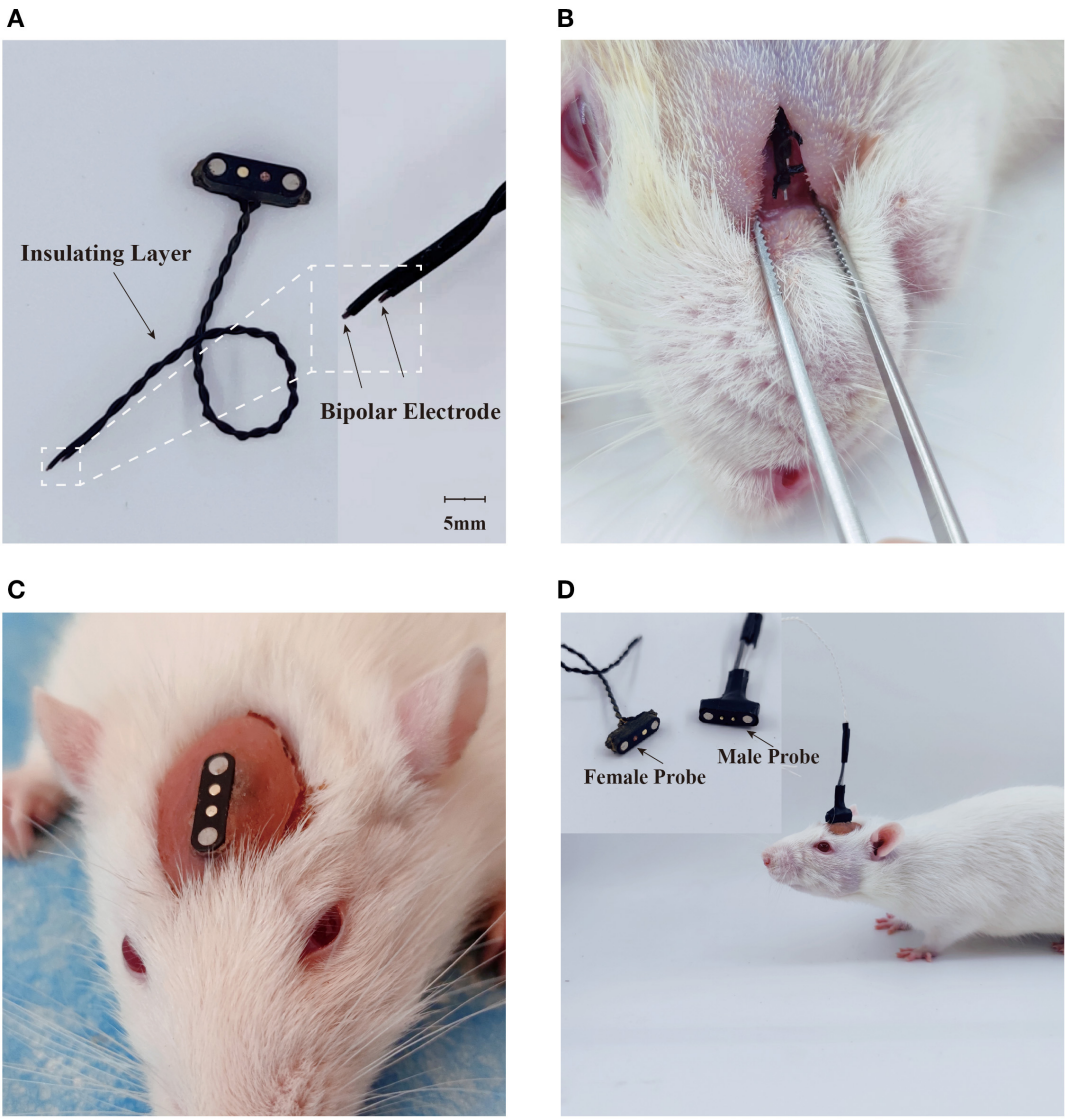
Design of electrical stimulation device. **(A)** Construction of electrical stimulator. **(B)** Placement of stimulation tetrode. **(C)** Implantation of electrical stimulation device. **(D)** Delivery of electrical stimuli in the freely-moving rodent.

One bipolar stimulation electrode was constructed by twisting two 200 μm diameter polyvinyl-chloride insulated tin-plated bronze wires (NO.30, Shenzhen KEBIWEI Company). The distal ending of the wire insulation was stripped 1 mm for electrical stimulation. One of the electrodes was cut 5 mm shorter than the other, leaving a space for the conduction of currents between the electrodes (Figure 2A). The proximal side of the electrode was welded to the female probe of one magnetic pogo pin (Shenzhen WEILICHUANG Electronic Company), which was connected to one electrical generator through the male probe of magnetic pogo pin.

### Device implantation

Rats were pre-anesthetized by inhalation in a sevoflurane (6–8%) anesthesia induction chamber for 1 minute, followed by maintenance of anesthesia with a sevoflurane (1.5–2.0%) mask and then placed at a stereotaxic holder. One median incision was performed and the soft tissue were removed to expose the surface of the skull. One subcutaneous tunnel was then made between the skull and the facial section, which was about 1.0 cm below the orbit of the eye. The stimulation side of the electrode was inserted through the tunnel and sutured subcutaneously as shown in the Figure 2B. After placement of electrode, the magnetic connector was secured to the skull screws with dental cement (Figures 2C,D). The animals were given about 14 days to perform any experiment after the surgical implantation.

### Stimulation protocol

Following the connection of the magnetic pogo pin, rats were placed in one transparent plastic cage (25^*^30^*^25 cm) to habituate for 10-15 minutes. The stimulation electrode was then connected with one direct current generator (Shenzhen Guce Electronic Company), by which we can adjust the intensity of electrical stimulation. A series of intensity was tested, ranging between 0 and 25V. To avoid the overreaction, the cut-off value of electrical intensity was set at 25V for the naïve rats and 16 for the ION-CCI group respectively. The behavioral video was continuously recorded at speed of 30 frames per second for further analysis.

### Response scoring criteria

To assess the electrical stimuli-evoked response, we modified previous response scoring criteria reported by Vos et al. (6). Specifically, the behavioral reactions following electrical stimuli were classified into three ascending categories: (1) detection, rat shows head movement upon the electrical stimuli, sometimes accompanied with exploratory sniffing behavior; (2) withdrawal or escape response, rat quickly moved its head and body following the stimuli and maintained one crouching gesture against cage wall, sometimes with its head buried under the body and made vocalizations, or escape response, either jumping or running. (3) asymmetric face grooming, we distinguished isolated face grooming from the movement during body grooming (19). If the episode was neither precede nor followed by body grooming (i.e., grooming patterns of a body area other than the face), the episode was considered as isolated grooming behavior. For each rat, one intensity was tested in one session, and repeated for five times. A mean score for each intensity was calculated to reveal the responsiveness.

### Mechanical allodynia test

As described previously (20–23), the well-established Dixon up-down method was applied to calculate the fifty percent withdrawal threshold, with a logarithmic series of Aesthesio Von Frey hairs (0.6, 1.0, 1.4, 2.0, 4.0, 6.0, 8.0, 10.0, 15.0 gram). Rats were habituated in the observation room for at least 20 minutes before testing. The Von Frey filaments were applied to the facial whisker pad of the rat to induce a brisk response of head withdraw (24). The interval between two trails should be kept above 30s.

### Conditioned place aversion

A two-chamber tool was used to conduct the CPA assay described as previously (21–23). The behavioral video in each chamber was continuously recorded by a high-resolution camera (Qianshiyan Technology Company, Shenzhen, China) at a speed of 30 frames per second. The CPA paradigm consisted of three consecutive 10-minute phases, including preconditioning, conditioning, and testing phases. Rats were free to access both chambers in the preconditioning session, those spent more than 480s in either chamber at baseline were excluded for further analysis. During conditioning phase, the electrical stimuli was applied to the facial region in one chamber every 10s, and none stimuli was paired with the other chamber. Animals did not receive any stimulation during testing phase, and had free access to both compartments. The recording of animal movement was retrospectively reviewed for the calculation of time spent in each chamber.

### Statistical analysis

Data were presented as mean ± standard error of the mean (SEM). We measured the normality of the variables with the Shapiro-Wilk testing. For response score testing, One-way or Two-way ANOVA followed by post-hoc multiple pair-wise comparison Bonferroni tests was used when appropriate. For the CPA testing, time spent in each chamber during the precondition phase was compared with that in the testing phase, with paired Student's *t*-test. Reduction of time spent in one chamber during testing session was associated with aversive response to the corresponding chamber. A CPA score was calculated by subtracting the time spent in the stimuli-paired chamber during the test phase from the time spent in that chamber at pre-conditioning phase (21–23). The CPA score was compared between naïve and ION-CCI rats with unpaired Student's test, one higher CPA score indicated enhanced aversive response to the noxious stimulation (23). *P*-value less than 0.05 was considered statistically significant. Statistical analysis was conducted by GraphPad Prism 8 (United States).

## Results

### Relationship between electrical stimuli intensity and sensory aspect of orofacial pain

**Figure 3:**
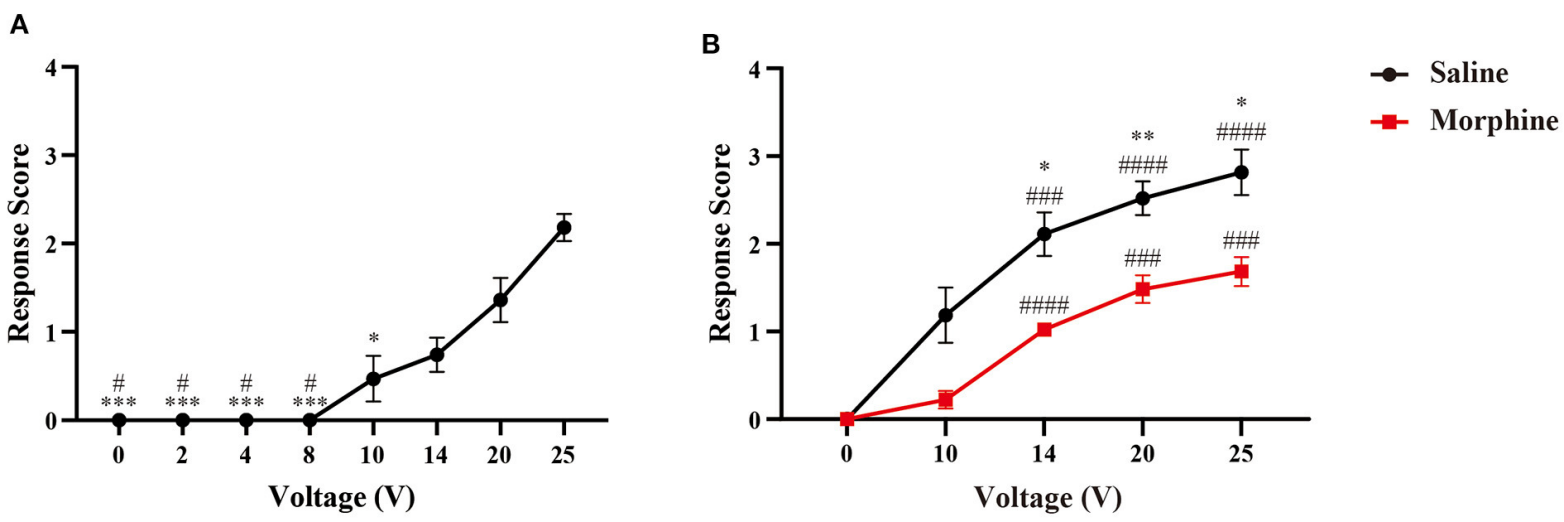
Responsive scoring testing in naïve rats. **(A)** Dose-response relationship between response scores and stimuli intensity. Data is presented as mean ± standard error of the mean (SEM). *N* = 10; *p < 0.05, ***p < 0.001, compared with 25V; ^#^*p* < 0.05, compared with 20V. One-way ANOVA with repeated measures and post-hoc Bonferroni test. **(B)** Analgesic effect of morphine on response scores. *N* = 9; *p < 0.05, **p < 0.01, morphine vs. saline; ^###^*p* < 0.001, ^####^*p* < 0.0001, intra-group analysis compared with 0V. Two-way ANOVA with repeated measures and Bonferroni's post-test.

We initially observed the evoked-response of electrical stimuli with a series of voltages (0, 2, 4, 8, 10, 14, 20, and 25 V). However, we did not observe any detection, withdrawal, escape or isolated facing grooming behaviors following electrical stimuli, with current voltage set below 10 volts. Only three of eleven rats displayed detection response when we increased the currents power to 10 volts. Response scores significantly increased with higher dose of electrical stimulation (20 and 25 V). About 60% (*n* = 6/10) rats displayed isolated grooming with 20V stimulation, and 64% for 25V respectively (Figure 3A).

### Analgesic effect of morphine on responsive reaction caused by electrical nociception

To evaluate the analgesic effect of morphine on nociceptive response caused by our device, we injected morphine or saline intraperitoneally before the behavioral testing. In Figure 3B, we can find that morphine treatment can attenuate the nociceptive response to the electrical stimuli. The response scores were reduced by the analgesic agent, as demonstrated by the significantly lower response scores at the voltages of 14, 20, and 25V compared with control group.

### Naïve rats represented an aversive response to the electrical stimulation with intensity of 25 V

**Figure 4:**
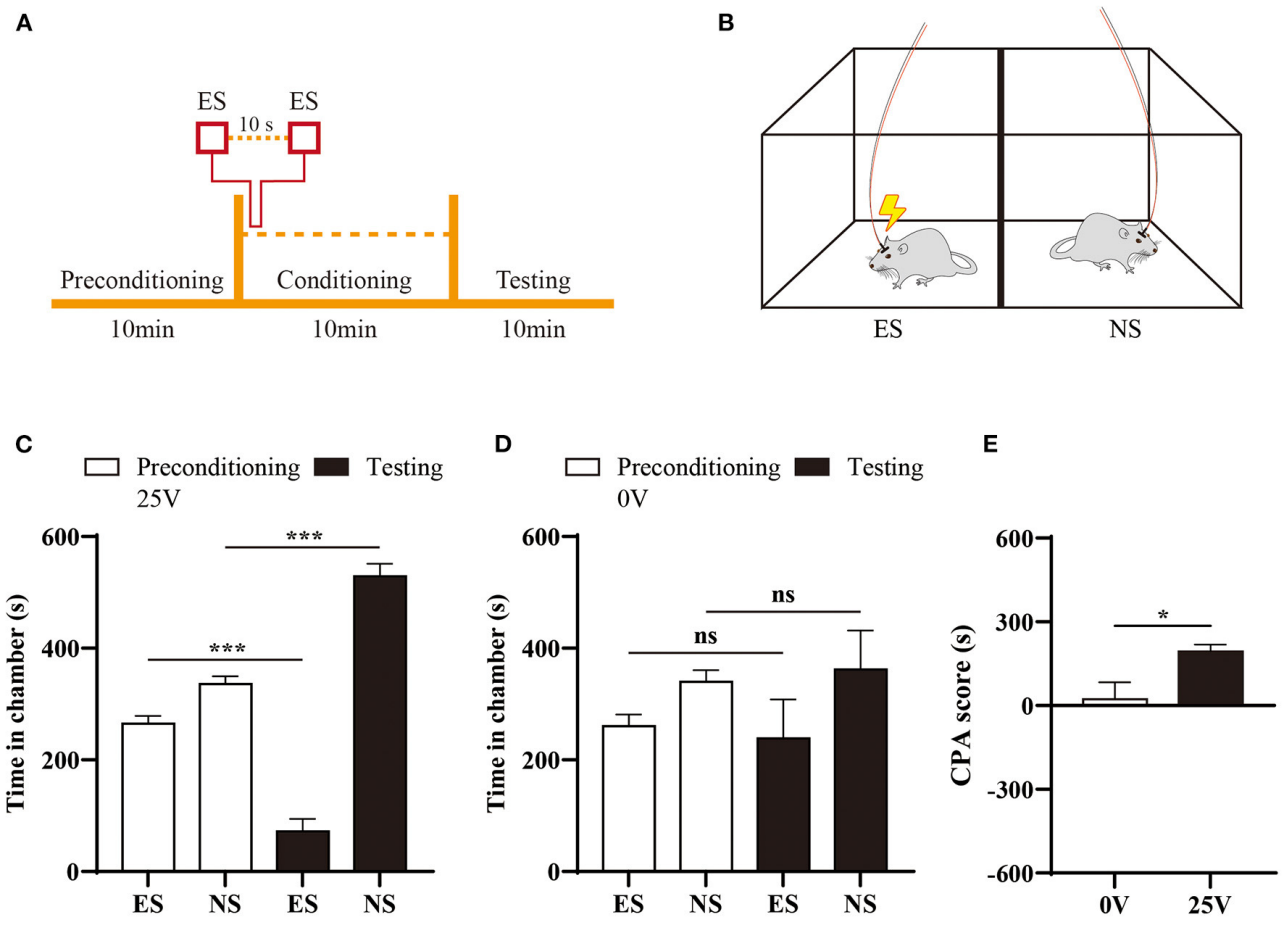
Aversive response to electrical stimulation of ION territory**. (A)** Schematic of CPA paradigm. **(B)** Rats received electrical stimulation (ES) in one chamber during conditioning phase of CPA, and none stimuli (NS) was paired with the other chamber. **(C)** Comparison of time spent in the chamber pre- and post-stimuli treatment. *N* = 6; ***p < 0.001, paired Student's *t*-test. **(D)** Sham stimuli did not induce preference to either chamber in the CPA testing. **(E)** Naïve rats developed aversion to 25V electrical stimuli of ION territory, demonstrated by an elevated CPA scores with sham stimuli treatment. *N* = 6; *p < 0.05, unpaired Student's *t*-test.

To evaluate the affective component of orofacial pain, we applied one well-established CPA paradigm in this study (21–23). During precondition phase, rats had free access to both chambers and showed no preference for either chamber. Next, we paired one chamber with electrical stimuli (25 V) at interval of 10s during conditioning phase (Figures 4A,B). In the testing session, time spent in the stimuli-paired chamber was significantly reduced compared with baseline (*p* < 0.001), as shown in Figure 4C. In contrast, rats did not develop avoidance to sham stimuli (Figure 4D). The difference of aversive response was further quantitated by an elevated CPA score in rats received 25V stimulation compared with control group (Figure 4E).

### Chronic orofacial pain induced sensory hypersensitivity to electrical stimuli

**Figure 5:**
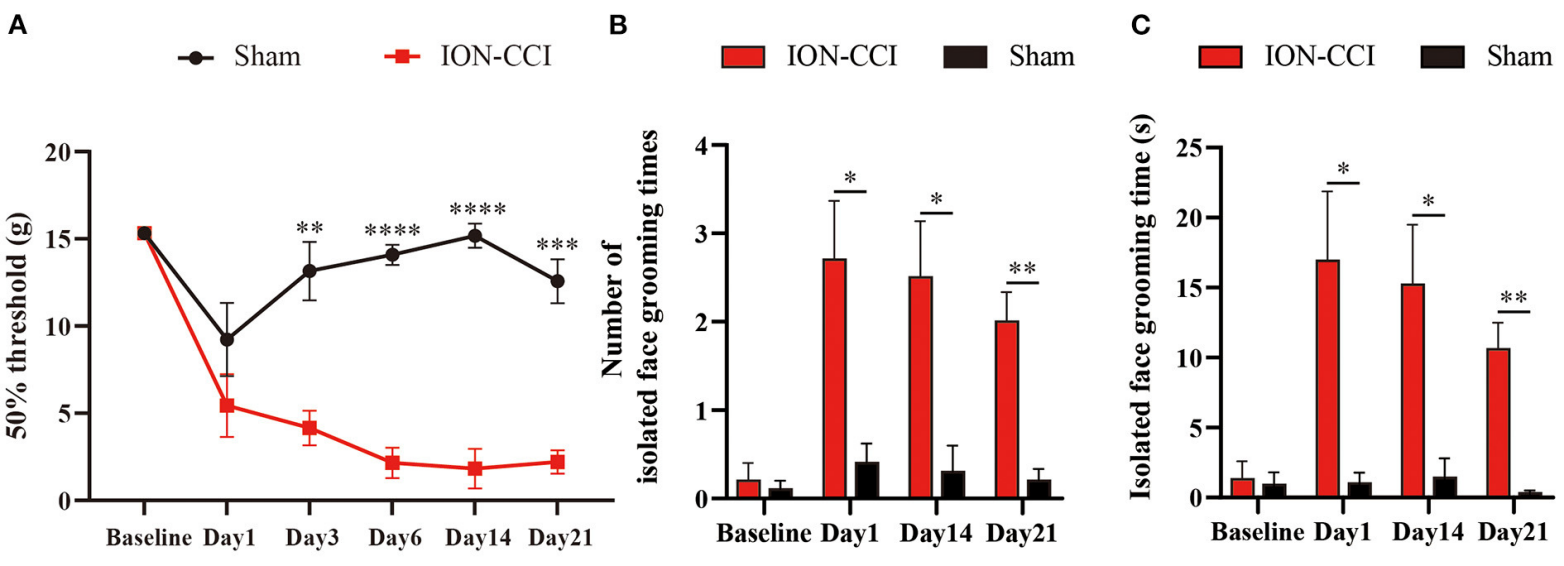
Chronic neuropathic orofacial pain induced sensory hypersensitivity. **(A)** Rats displayed mechanical allodynia after establishment of ION-CCI. *N* = 6; **p < 0.01, ***p < 0.001, ****p < 0.0001, Two-way ANOVA with repeated measures and Bonferroni's post-test. **(B,C)** ION-CCI Rats developed spontaneous pain-like behavior, as demonstrated by increasing isolated grooming behavior. *N* = 10; *p < 0.05, **p < 0.01, Two-way ANOVA with repeated measures and Bonferroni's post-test.

Next, we tested our device in rats with neuropathic orofacial pain (ION-CCI model). Chronic constriction of ION significantly increased sensory sensitivity, demonstrated by mechanical allodynia (Figure 5A). In addition to evoked hypersensitivity, we also observe spontaneous pain-like behaviors in ION-CCI model. In Figures 5B,C, we show that neuropathic orofacial pain induced more isolated grooming episodes than control group.

**Figure 6:**
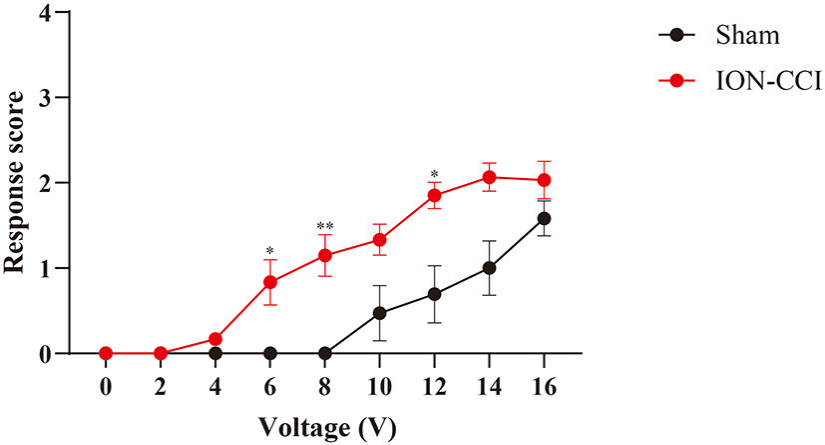
Comparison of response scores between ION-CCI rats and sham group. Neuropathic orofacial pain induced sensory hypersensitivity to electrical stimuli, demonstrated by an increasing response score compared with naïve rats. *p < 0.05, **p < 0.01, Two-way ANOVA with repeated measures and Bonferroni's post-test.

To capture electrical stimuli-induced response in neuropathic orofacial pain state, we implanted electrical stimulator before establishment of ION-CCI model. The responsive threshold of electrical stimuli became significantly lower after neuropathic lesion compared with baseline (Figure 6). Five of ten rats displayed detection movement following 4V electrical stimuli. In ION-CCI rats, withdrawal, escape and isolated grooming can be induced by electrical stimulation at voltage of 6V, which naïve rats did not demonstrate any noticeable reaction. The threshold to reach maximal response score also decreased in chronic orofacial pain condition. Thus, we set cut-off value of stimuli intensity at 16V to avoid discomfort of animal.

### Aversion of orofacial pain was enhanced in ION-CCI model

**Figure 7:**
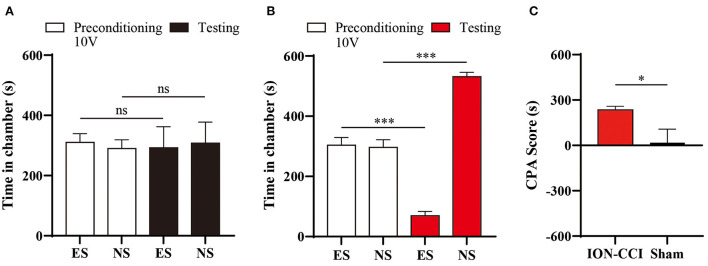
Enhancement of aversion to electrical stimuli induced by neuropathic orofacial pain. **(A)** Sham group rats did not display significant avoidance to the chamber paired with 10V electrical stimuli (ES). **(B)** ION-CCI rats demonstrated significant aversive response to the chamber paired with ES during the testing phase during the CPA experiments. ***p < 0.001, paired Student's *t*-test. **(C)** Comparison of CPA score between ION-CCI and sham group. *p < 0.05, unpaired Student's *t*-test.

In our previous study, we found that chronic pain enhanced aversive response to mechanical nociception (23, 25). We thus investigated the aversive effect of electrical stimulation in ION-CCI rats, by pairing one chamber with 10 volts electrical stimulation during conditioning phase of CPA testing (Figure 7A). We found that avoidance of stimuli-paired chamber was developed in ION-CCI rats (Figure 7B), but not in sham group (Figure 7C).

## Discussion

Testing the sensory and affective contents of orofacial pain in animal subjects remains a challenge. In this study, we showed one novel implantable device to delivery electrical nociception to naïve and neuropathic pain rat, to quantitively evaluate the sensory and affective components of orofacial pain. Thus, our finding provides an alternative option for basic research of orofacial pain.

Currently, majority of extant animal models of orofacial pain have been developed to mimic a specific neuropathic phenotype, which do not necessarily match the clinical manifestations of trigeminal neuralgia (26). One limitation is the lack of clinically relevant symptoms, such as stimulus-evoked pain reported in 99% of classic trigeminal neuralgia patients (27). One well-established response scoring tool has been widely applied to assess sensory hypersensitivity to mechanical stimuli in the territory of ION (6). However, the manual operation of stimulation delivery to the facial region remains a difficult task in animal study. In this study, we tried to solve this technical issue by implanting the stimuli device percutaneously and applying electrical stimulation remotely. Consequently, the behavioral responses to stimuli were not disturbed or affected by the motion of operator with this novel instrument.

In addition to manual interference, another obvious advantage of this device is the adjustable and stable design of stimuli output. Even for the same operator, the actual mechanical threshold may be different across multiple trials, due to distinct stimuli site, approaching access, and duration of stimulation with Von Frey filaments (6). In this study, the parameter of stimulation such as current voltage and interval period, can be well controlled and adjusted according to the responsiveness of animal. Thus, a series of current intensity was applied to test the evoked reaction, ranging from 2 to 25V. To avoid excessive stimulation, we set cut-off value of stimulation intensity at 25V for naïve rats, and 16V for ION-CCI subjects respectively.

Consistent with mechanical testing, we found that higher order-rank response content was induced by increasing intensity of stimulation. Specifically, we did not observe any evoked reaction in naïve rat with current power under 10V. Detection, withdrawal, or escape can be induced when the stimuli current was set above 10V in naïve rats (Figure 3A). The asymmetric face grooming is considered as a prolonged aversive response, which was more commonly found in the session with higher intensity (20 and 25V). Despite current intensity, it has been demonstrated that frequency of electrical stimulation may have an impact on craniofacial pain (15). However, the mechanism underlying the nociceptive reflex induced by peripheral stimulation may be different from that targeted dura mater.

Hyperalgesia is frequently reported in chronic pain state, which is defined as increased pain from a stimulus that normally provokes pain (28). In animal study, multiple types of nociceptive stimuli (electrical, thermal, mechanical, or chemical) have been used in different pain model (29, 30). Despite pain research, electrical stimuli have also been widely used in testing of fear conditioning, learned helplessness, and aversion protocol (31–33). To quantitively measure the electoral thresholds, electrical stimuli is given at given intervals, with a fixed increment in intensity for both animal and human being (34, 35). In current study, we applied square-wave electrical stimulation with a one-second duration, which is more relevant to clinical manifestation that directly excites full spectrum of peripheral nociceptors in trigeminal neuralgia cases. However, we should be aware of that an unnatural and non-specific electrical stimulation may not only reveal the sensory information, but also a nociceptive response.

To capture the affective component of pain with this novel device, we further administrated electrical stimuli in one well-established CPA protocol (21–23). According to the response scoring system (6), asymmetric face grooming indicated a prolonged aversive response following facial nociception. Thus, we chose to test the aversive effect of 25V electrical stimuli, with which we can frequently observe isolated grooming behavior (Figure 3). We paired one chamber with 25V electrical stimuli in naïve animal, none stimuli were given in the other chamber during the conditioning phase of CPA testing (Figure 4). The aversive response to electrical stimulation was demonstrated by significant reduction of time spent in the stimuli-paired chamber.

Consistent with previous report, ION-CCI rats exhibited hypersensitivity to both innoxious and noxious stimuli (6, 36). A lower response threshold to electrical stimuli (4V) was found in ION-CCI rats compared with sham group, which did not display any evoked-behavior up to 10V stimulation (Figures 3, 6). The CPA data also supports the development of hypersensitivity in chronic neuropathic orofacial pain state, that a mild stimuli intensity (10V) induced a significant avoidance in ION-CCI rather than control group (Figure 7). The phenomenon of aversive enhancement is consistent with our previous results in inflammatory pain model (23, 25). Thus, our findings suggest one potential tool for hypersensitivity assessment of sensory and affective orofacial pain.

It has been thought that pathological condition of neuropathic orofacial pain can induce the hyperactivity of the neurons in the trigeminal ganglion, which receive the peripheral noxious input and then project to the higher order of the central nerve system, including trigeminal spinal subnucleus caudalis, thalamic nucleus, somatosensory, and limbic regions (37, 38). In addition to this classic neural pathway of orofacial pain processing, recently, the dysfunction of the lateral parabrachial nucleus and its projection to the emotion-related limbic regions has been demonstrated to contribute to the augment of the orofacial pain (4). As a result, the peripheral and central sensitization at distinct level above may contribute to the generalized enhancement of sensory and affective orofacial pain.

There are several limitations in this study. First, we only tested the analgesic effect of morphine on the nociceptive response induced by the electrical stimulator. We think it necessary to test other analgesic agents like local anesthetics in the future. Additionally, the dosage effect of analgesic agents also remains to be further investigated. Likely, we only tested our device in the neuropathic pain model, the usage in other phenotype of pathological orofacial pain (e.g., inflammatory pain or headache disorder) is also needed to be considered.

## Conclusion

It is feasible to apply the implantable device for delivery of electrical stimulation in the facial region. The evoked-response of rats following electrical stimuli was induced in one dose-dependent manner. In addition, ION-CCI rats demonstrated both sensory and affective hypersensitivity compared with naïve group. Our novel device may provide an alternative option for measurement of sensory and affective dimension of orofacial pain.

## Data availability statement

The original contributions presented in the study are included in the article/supplementary material, further inquiries can be directed to the corresponding authors.

## Ethics statement

The protocol of animal experiment was approved by the Institutional Animal Care and Use Committee, Central South University.

## Author contributions

HZ, XY, and DH designed the experiments. ZL, XH, and JM performed the implantation and ION-CCI surgery. XH, ZL, and JM conducted the behavioral testing. XH and ZL did the statistical analysis. HZ wrote the manuscript. All authors contributed to the article and approved the submitted version.

## Funding

This research was funded by National Natural Science Foundation of China, (81901146 to HZ and 82071140 to HZ and XY), the Key Laboratory of Hunan Province Grants (2018TP1009 to HZ and DH), Excellent Youth Foundation of Hunan Scientific Committee, and the Huizhiyucai Project of the Third Xiangya Hospital, Central South University.

## Conflict of interest

The authors declare that the research was conducted in the absence of any commercial or financial relationships that could be construed as a potential conflict of interest.

## Publisher's note

All claims expressed in this article are solely those of the authors and do not necessarily represent those of their affiliated organizations, or those of the publisher, the editors and the reviewers. Any product that may be evaluated in this article, or claim that may be made by its manufacturer, is not guaranteed or endorsed by the publisher.
